# Fibroblast Growth Factor-2 Enhances Expansion of Human Bone Marrow-Derived Mesenchymal Stromal Cells without Diminishing Their Immunosuppressive Potential

**DOI:** 10.4061/2011/235176

**Published:** 2011-03-03

**Authors:** Jeffery J. Auletta, Elizabeth A. Zale, Jean F. Welter, Luis A. Solchaga

**Affiliations:** ^1^Divisions of Pediatric Hematology/Oncology and Pediatric Infectious Diseases, Department of Pediatrics, University Hospitals Case Medical Center, Cleveland, OH 44106, USA; ^2^Hathaway Brown School, 19600 North Park Boulevard, Shaker Heights, OH 44122, USA; ^3^Skeletal Research Center, Department of Biology, Case Western Reserve University, Cleveland, OH 44106-7080, USA; ^4^Division of Hematology and Oncology, Department of General Medical Sciences, Case Western Reserve University School of Medicine, Cleveland, OH 44106, USA; ^5^BioMimetic Therapeutics, Inc., Franklin, TN 37067, USA

## Abstract

Allogeneic hematopoietic stem cell transplantation is the main curative therapy for many hematologic malignancies. Its potential relies on graft-*versus*-tumor effects which associate with graft-*versus*-host disease. Mesenchymal stromal cells (MSCs) possess immunomodulatory properties that make them attractive therapeutic alternatives. We evaluated the *in vitro* immunosuppressive activity of medium conditioned by human MSCs from 5 donors expanded 13 passages with or without FGF-2. FGF-2 supplementation increased expansion 3,500- and 240,000-fold by passages 7 and 13, respectively. There were no differences in immunosuppressive activity between media conditioned by passage-matched cells expanded under different conditions, but media conditioned by FGF-treated MSCs were superior to population doubling-matched controls. The immunosuppressive activity was maintained in three of the preparations but decreased with expansion in two. The proliferation induced by FGF-2 did not result in loss of immunosuppressive activity. However, because the immunosuppressive activity was not consistently preserved, caution must be exercised to ensure that the activity of the cells is sufficient after extensive expansion.

## 1. Introduction

Allogeneic hematopoietic stem cell transplantation (HSCT) is the only curative therapy for many hematologic malignancies. Part of the therapeutic benefit of allogeneic HSCT is based on graft-*versus*-tumor (GVT) effects, which eradicate residual malignant cells through immunologic mechanisms. Unfortunately, GVT is closely associated with the development of graft-*versus*-host disease (GVHD) [1], which is one of the primary causes of transplant-related mortality [2]. Less than half of patients that develop severe acute GVHD respond to first-line corticosteroid therapy [3, 4]. Patients with steroid-resistant GVHD require secondary therapy to which only half of these patients respond and have overall poor survival [2, 5]. Therefore, new therapeutic approaches to prevent and treat GVHD are urgently needed. Cellular therapies are emerging as promising approaches to treat and even to prevent immune-aberrant diseases such as GVHD.

Mesenchymal stromal cells (MSCs) are nonhematopoietic multipotent cells capable of differentiating into both mesenchymal and nonmesenchymal lineages [6–8]. MSCs also produce cytokines, chemokines, and extracellular matrix proteins that support *in vitro* hematopoietic stem cell (HSC) survival and proliferation and facilitate *in vivo* HSC engraftment [9]. A substantial body of evidence suggests that MSCs are capable of inhibiting T-lymphocyte activation and proliferation *in vitro *[10–14]. Additionally, MSCs appear to be immunoprivileged and to escape immune surveillance eliciting only weak responses upon rechallenges [15, 16]. These characteristics make MSCs very attractive as cellular therapeutic agents [17, 18]. 

Human MSC (hMSC) preparations have significant, though variable and finite, proliferative potential [19]. This variability may be due to exogenous factors such as the method used to obtain the bone marrow [20–24], specifics of the serum lots used, and intrinsic factors such as the age of the donor [21, 25]. Despite their high proliferative potential, extensive culture expansion may result in loss of differentiation potential and the onset of senescence [21]. Interestingly, the senescence-related loss of potential is not generalized; for example, MSCs maintain their osteogenic potential through extensive subcultivation [20, 21], but lose their ability to differentiate into adipocytes [21] as they approach senescence. Their ability to differentiate into chondrocytes is lost in earlier passages [26]. 

Several published reports have demonstrated that fibroblast growth factor-2 (FGF-2) exerts a significant mitogenic effect on MSCs while, simultaneously, enhancing their tri-lineage (bone, cartilage, fat) differentiation capacity [27–32]. Chondrogenesis, perhaps the differentiation potential most easily lost in MSCs during *in vitro* culture, is particularly improved by expansion in FGF-2-supplemented medium [30, 31].

Little is known, however, about the maintenance or loss of the immunomodulatory activity of hMSCs through extensive expansion or the impact that FGF-2 supplementation might have on this MSC property. The purpose of these experiments was to characterize the immunosuppressive activity of MSCs expanded *in vitro* for different periods of time with and without FGF-2 supplementation which, as stated above, has been shown to be beneficial for the maintenance of other hMSC functions.

## 2. Material and Methods

All cells were isolated from normal healthy human donors at the Hematopoietic Stem Cell Core Facility of the Comprehensive Cancer Center of Case Western Reserve University after informed consent was obtained under the terms of an Institutional Review Board-approved protocol. Peripheral blood mononuclear cells (PBMCs) were isolated from peripheral blood collected into heparinized blood collection tubes (BD, Franklin Lakes, NJ). Human MCSs were isolated from bone marrow aspirates obtained from the posterior superior iliac crest into a preheparinized 20-mL syringe (BD). The PBMCs and hMSCs used in these studies were isolated from different, unrelated donors.

### 2.1. Isolation of Human PBMCs

Ten human PBMC preparations were used in these studies. The blood was carefully layered on top of Ficoll (GE Healthcare, Pisctatway, NJ) and the tubes centrifuged at 800 ×g for 30 minutes without brake. After centrifugation, a sterile plastic pipette was used to aspirate the mononuclear cell layer and transfer it into a fresh 50 mL conical tube (BD). The PBMCs were then washed twice with phosphate buffered saline (PBS, Invitrogen, Carlsbad, CA), counted, and resuspended in complete Roswell Park Memorial Institute (RPMI) 1640 medium composed of RPMI 1640 (Invitrogen) supplemented with 10% heat-inactivated fetal bovine serum (FBS, Invitrogen).

### 2.2. Isolation of Human MSCs

Five hMSC preparations were used in this study. The procedures for establishing human bone marrow-derived MSC cultures followed previously published methods [33, 34]. Briefly, bone marrow aspirates were washed with control medium consisting of low glucose Dulbecco's modified Eagle's medium (DMEM-LG, Invitrogen) supplemented with 10% fetal bovine serum (FBS) from a selected lot (Hyclone, Logan, UT) [34]. Serum lot selection is a standard procedure performed prior to purchasing a new shipment of serum; all experiments were conducted with serum from a single lot. They were then centrifuged on a Percoll (Sigma Chemical Co., St. Louis, MO) density gradient to isolate mononuclear cells. The mononuclear cells were washed with control medium and seeded at a density of 1.8 × 10^5^ cells/cm^2^ in control medium to establish primary cultures. All cell culture was done at 37°C in a humidified atmosphere of 95% air and 5% CO_2_.

### 2.3. Establishment of Study Groups

At the first medium change (day 4), and in every medium change thereafter, some of the plates received control medium, and the rest of the plates received the same medium supplemented with 10 ng/mL of FGF-2 (Peprotech, Rocky Hill, NJ). The dose was chosen based on previous studies [31]. Cultures were fed twice per week.

### 2.4. Expansion of hMSCs

hMSCs must be subcultured before the cells become confluent in order to keep their growth at an exponential rate and prevent spontaneous differentiation or loss of differentiation potential [6, 35]. Typically, they were passaged when the cultures were 80−90% confluent. Primary cultures were usually subcultured at 14 ± 3 days. Subsequently, the cells were subcultured approximately every 7 ± 2 days. Plates assigned to the different study groups (control or FGF-treated) were subcultured at the same time, which resulted in different levels of confluence in the treatment groups as result of the previously reported differences in cell proliferation and cell size [31]. In all cases, control cultures were less confluent than their FGF-treated counterparts at the time of subcultivation. Cells were subcultured by trypsinization, counted, and reseeded at 4.5 × 10^3^ cells per cm^2^.

### 2.5. Cryopreservation of hMSCs

Subcultured cells were centrifuged at 200 ×g for 5 minutes; the supernatant was discarded, and the cells resuspended in cold freezing medium consisting of 90% FBS (Invitrogen) and 10% DMSO (Sigma Chemical Co.) at a density of 10^6^ cells/mL. The cells were aliquoted into cryogenic storage vials (Thermo Fisher Scientific, Rochester, NY), the vials placed in a freezing container (Thermo Fisher Scientific), and the container placed at −80°C overnight. The vials were then transferred to the vapor phase of a liquid nitrogen freezer.

### 2.6. Recovery of Cryopreserved hMSCs

Vials containing approximately 1.0 − 1.5 × 10^6^ cryopreserved cells were thawed quickly in a 37°C water bath and transferred into a 15-mL conical tube containing 5 mL of either control or FGF-supplemented medium, as appropriate. The tubes were then centrifuged at 200 ×g for 5 minutes. After centrifugation the supernatant was discarded, and the cells were resuspended in either control or FGF-supplemented medium and seeded at 4.5 × 10^3^ cells per cm^2^.

### 2.7. Characterization of hMSCs

The hMSC phenotype was confirmed by forward and side-scatter pattern as well as monoclonal antibody (MAb) staining (CD45^−^, CD73^+^, CD105^+^). Briefly, approximately 500,000 hMSCs were washed and resuspended in HBSS supplemented with 1% FBS containing 5 mM EDTA. hMSCs were then incubated with fluorescein isothiocyanate (FITC)-conjugated anti-CD45 MAb (BD), phycoerythrin (PE)-conjugated anti-CD73 MAb (BD), and allophycocyanin (APC)-conjugated anti-CD105 MAb (eBioscience, San Diego, CA). After 30-minute incubation at 4°C, MAb-labeled cells were washed three times and then fixed in 2% paraformaldehyde prior to analysis. Isotype control immunoglobulins were used as negative controls for staining. At least 20,000 events were analyzed per conjugated MAb stain condition. 

To assess their chondrogenic potential [36–38], passaged cells were resuspended in a chemically defined chondrogenic medium. Aliquots containing 2.5 × 10^5^ cells were placed in polypropylene multiwell plates, centrifuged at 500 ×g, and placed in the incubator. Medium was changed three times per week. On days 7, 14, and 21 aggregates were harvested and processed for histologic evaluation.

To verify their osteogenic potential [20, 39, 40] hMSCs were seeded at a density of 5 × 10^3^ cells/cm^2^ in control medium and allowed to attach overnight. The next day, the culture medium was replaced with osteogenic medium. Culture medium is changed twice per week. Osteogenic cultures are further supplemented with 2 mM *β*-glycerophosphate starting on day 10. Triplicate cultures were stained for calcium deposition (von Kossa) [41] on days 14, 21, and 28.

The adipogenic potential of hMSCs was also tested [42]; passaged cells aliquots containing 2.5 ×10^5^ cells were placed in polypropylene multiwell plates in adipogenic induction medium in multiwell plates, centrifuged at 500 ×g, and placed in the incubator. Medium was changed three times per week. On day 10, the medium was replaced with adipogenic maintenance medium. Cells were harvested for histologic analysis on days 7, 14, and 21.

### 2.8. Generation of hMSC-Conditioned Media

hMSCs at different passage numbers were seeded into 6-well plates (BD), at a density 15 × 10^3^ cells/cm^2^ in complete hMSC medium. After overnight incubation, the medium was removed and a 2-mL aliquot of complete RPMI containing either PBMCs (0.5 × 10^6^ cells/mL) or interleukin-1 beta (IL-1*β*) (5 pg/mL, Peprotech) was added to each well. Control wells received only complete RPMI. Another set of control wells was incubated without hMSCs, but still received either PBMCs or IL-1*β*. After a 24-hour incubation period, the conditioned media were collected into 2-mL microcentrifuge tubes and centrifuged for 10 minutes at 12,000 ×g to remove any remaining cells. The cell-free supernatants were then transferred to clean microcentrifuge tubes and either used fresh or frozen at −80°C for later use.

### 2.9. Assessment of Cell Expansion

All cell counts during the expansion phase were done manually on the trypsinized cell suspensions, in triplicate using a Neubauer hemocytometer. Population doublings were calculated as the base-2 logarithm of the number of cells obtained at the end of a given passage divided by the number of cell seeded.

For primary cultures, the number of colonies formed in primary culture we used as the denominator assuming that one colony is derived from one MSC.

### 2.10. Interferon-Gamma ELISpot Assay

The immunosuppressive activity of hMSCs and their conditioned media was tested in enzyme-linked immunosorbent spot assays (ELISpot) [43]. The ELISpot assay allows visualization of the secretory product of individual responding cells; each spot that develops in the assay represents a single reactive cell. Thus, the assay provides both qualitative (type of immune protein) and quantitative (number of responding cells) information. ELISpot assays are highly sensitive because the product is rapidly captured around the secreting cell before it is diluted in the supernatant, captured by receptors of adjacent cells, or degraded. The assay has gained a recent increase in popularity, especially as a surrogate measure for cytotoxic T-cell responses in large part because it is both reliable and highly sensitive [44].

Ninety-six well ELISpot plates (Millipore, Billerica, MA) were coated with antihuman interferon-gamma (IFN*γ*) antibody (Pierce, Rockford, IL); 100 *μ*L of antibody solution (4 *μ*g/mL in PBS) were added to each of the 96 wells of the plate and incubated overnight in the refrigerator. The plate was then washed with PBS and blocked at 37°C for 2 hours with complete RPMI. The wells then received either 150 *μ*L of complete RPMI (control wells) or 150 *μ*L of either a 10^6^ cells/mL hMSC suspension or hMSC-conditioned medium (experimental wells). Then, 25 *μ*L of complete RPMI were added to the negative control wells, and 25 *μ*L of phytohemagglutinin (PHA, Sigma Chemical Co) solution (40 *μ*g/mL in complete RPMI) were added to experimental and positive control wells. A 25-*μ*L aliquot of PBMC suspension (6 × 10^6^ cells/mL) was finally added to each well, and the plate was incubated for 24 hours at 37°C. After the incubation, the plate was washed with PBS + 0.05% Tween 20 (Sigma Chemical Co.). Biotinylated anti-IFN*γ* antibody (Pierce) (2 *μ*g/mL in PBS + 0.05% Tween 20 + 1% BSA (Sigma Chemical Co.) was added and the plate incubated at 37°C for 2 hours. After washing the plate with PBS + 0.05% Tween 20, Streptavidin-Horseradish Peroxidase (Dako, Glostrup, Denmark) diluted 1 : 1,000 in PBS + 0.05% Tween 20 + 1% BSA was added and the plate incubated for 1 hour. After 3 washes with PBS + 0.05% Tween 20 followed by 4 washes with PBS the IFN*γ*-positive spots were developed with 3-amino-9-ethyl carbazole (AEC) (Pierce). The reaction was then stopped with tap water, and the plates were allowed to dry in the dark. The plates were analyzed with a computer-assisted ELISpot analyzer (Cellular Technology Inc., Cleveland, OH). Percent inhibition was obtained by direct comparison to the corresponding positive control indicated above.

### 2.11. Statistical Analyses

The statistical significance of the differences in proliferation rates and of the differences between PBMC and IL-1*β* stimulation was determined by paired *t*-tests. Analysis of the effect of time-in-culture on the immunosuppressive activity was performed by one-factor repeated measurements ANOVA followed by paired *t*-tests to identify the differences between passages. The analysis of the effect of FGF supplementation was performed by paired *t*-tests, and differences were considered significant for *P* values <.05.

## 3. Results

### 3.1. Cell Expansion

**Figure 1:**
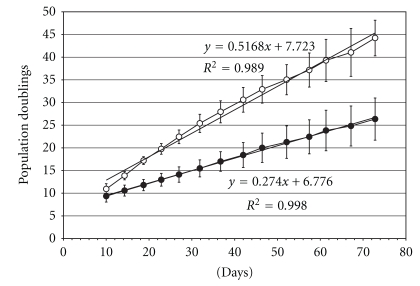
Culture Expansion. Cumulative population doublings of hMSCs expanded in control conditions (black circles) or in the presence of rhFGF-2 (white circles). Mean ± standard deviation from 5 individual hMSC preparations. The slopes of the linear regression equations are an approximation to the average population doubling times.

As in previous studies, hMSCs expanded in the presence of FGF-2 exhibited higher proliferation rates than those expanded in control conditions [31]. The population doubling time for hMSCs expanded in the presence of FGF-2 was consistently shorter than that of cells expanded under control conditions at any passage (*P* < .01) as indicated by the slope of the growth curves in Figure 1. By passage 13, control MSCs reached on average 26.3 ± 4.7 population doublings (PDs), while FGF-treated cells reached 44.2 ± 3.9 PDs. FGF-treated hMSCs reached 28.0 ± 2.6 PDs (the maximum expansion obtained with control cells) around passage 6 in approximately 35 days. Control or FGF-treated cells were subcultured at the same time, causing different levels of confluence, a consequence of the differences in cell proliferation and cell size [31]. Control cultures were never more confluent than their FGF-treated counterparts at any time of subcultivation.

### 3.2. Cell Characterization

**Figure 2:**
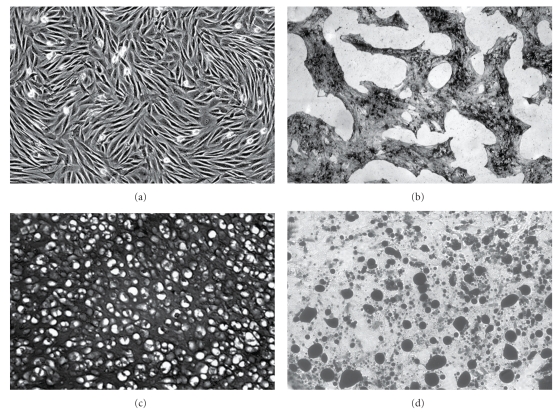
Functional characterization of hMSCs. Human MSCs (a) in culture, (b) after 4 weeks in osteogenic conditions (von Kossa staining), (c) after 3 weeks in chondrogenic conditions (toluidine blue staining), and (d) after 3 weeks in adipogenic conditions (oil-red O staining).

hMSCs isolated from bone marrow exhibited the typical characteristics of multipotent hMSCs [45], that is, characteristic morphology, surface marker profile (not shown), and trilineage differentiation potential (Figure 2).

### 3.3. Assessment of the Immunomodulatory Activity of hMSC-Conditioned Medium

**Figure 3:**
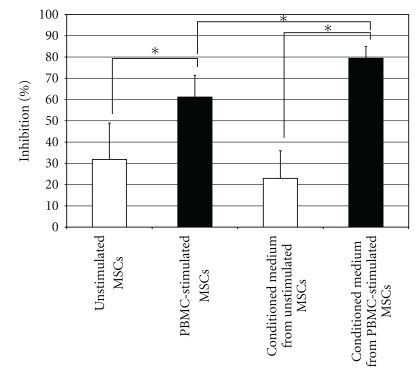
Immunosuppressive activity of hMSC-conditioned medium. Immunosuppressive activity of unstimulated (white bars) and PBMC-stimulated (black bars) hMSCs and hMSC-conditioned media. Values are expressed as percent inhibition relative to PHA-stimulated PBMCs which served as controls. Higher bars indicate more immunosuppressive activity. Asterisks indicate significant differences in one-tail *t*-test; *P < .01.

Conditioned medium from PBMC-stimulated hMSCs exhibited higher activity than the PBMC-stimulated hMSCs themselves (one-tail *t*-test; *P* = 5.52 × 10^−7^; *n* = 5), as measured by decreases in the number of IFN*γ*-positive spots. The immunosuppressive potential of PBMC-stimulated hMSCs and their conditioned media was higher (one-tail *t*-test; *P* = 1.46 × 10^−7^; *n* = 5) than that of the conditioned media from nonstimulated hMSCs and the non-stimulated hMSCs themselves (Figure 3). Supported by these results and for logistical reasons and ease of use, in-depth analysis of the immunosuppressive activity of hMSCs from the study groups at different passages was performed on conditioned media samples rather than with the cells.

### 3.4. Stimulation of hMSCs by IL-1*β*

**Figure 4:**
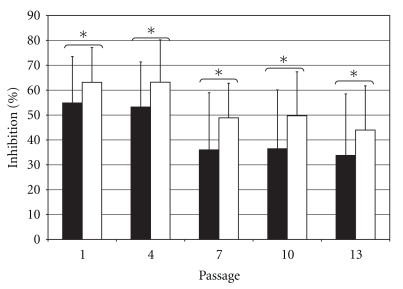
Effect of PBMC and IL-1*β* stimulation on the immunosuppressive activity of hMSCs. Immunosuppressive activity (mean ± standard deviation; *n* = 10; 5 control and 5 FGF-treated) of medium conditioned by MSCs from different passages stimulated with PBMCs (black bars) or IL-1*β* (white bars). Values are expressed as percent inhibition relative to the internal controls. Higher bars indicate higher immunosuppressive activity. Asterisks indicate significant differences in one-tail paired *t*-test; *P < .05.

Conditioned media generated after activation of hMSCs with either PBMCs or IL-1*β* exhibited immunosuppressive activity in IFN*γ* ELISpots (Figure 4). The conditioned media generated by stimulation of hMSCs with IL-1*β* exhibited higher (one-tail paired *t*-test; *P* = 5.23 × 10^−7^, *n* = 50) and more consistent immunosuppressive activity (CV = 33%) than media from cultures stimulated with PBMCs (CV = 54%). 

The higher variability of the conditioned media generated by stimulation of hMSC cultures with PBMCs likely reflects the interindividual variability among the blood donors from whom the PBMCs were isolated.

Consequently, for simplicity and data consistency, we focused our analyses on conditioned media from IL-1*β*-stimulated hMSCs although both PBMC and IL-1*β* stimulation were tested for all cultures.

### 3.5. Effect of Ex Vivo Expansion on the Immunosuppressive Potential of hMSCs

To analyze the effect of time-in-culture on the immunosuppressive potential of each hMSC preparation, the inhibitory activity of each passage was normalized to that of the first passage of that preparation to minimize variability in the intrinsic degree of inhibition among the 5 cell preparations.

**Figure 5:**
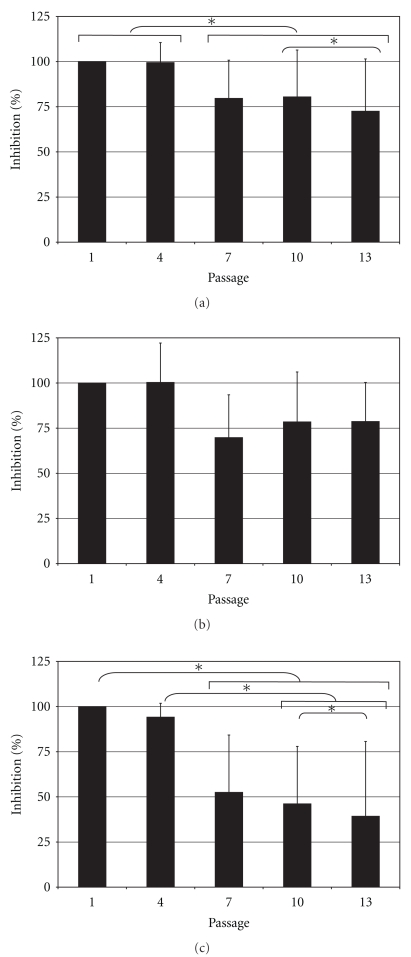
Effect of time-in-culture on the immunosuppressive activity of hMSCs. Immunosuppressive activity in conditioned medium from IL-1*β*-stimulated hMSCs at different passages cultured under control conditions. Values are normalized to the inhibition observed conditioned medium from first passage hMSCs. The mean and standard deviation for all 5 hMSC preparations are displayed in (a); the 3 hMSC preparations that exhibited sustained activity are displayed in (b); the mean and standard deviation for the 2 hMSC preparations that exhibited decreased activity after 4 passages are displayed in (c). Asterisks indicate significant differences in one-tail paired *t*-test; *P < .05.

Overall, when all five cell preparations were considered, there was a decrease in the immunosuppressive activity as a function of time in culture (single factor ANOVA; *P* = .010, *n* = 10) (Figure 5(a)). Conditioned media from cells at passage 1 and passage 4 had equivalent activity (one-tail paired *t*-test; *P* = .437, *n* = 10) to one another and superior to the activity of conditioned media from cells at passages 7, 10, and 13 (one-tail paired *t*-test; *P* ≤ .044,   *n* = 10); conditioned media from cells at passage 7 had similar activity to that of cells from passages 10 and 13 (one-tail paired *t*-test; *P* ≥ .146,   *n* = 10), and conditioned media from cells at passage 10 were more immunosuppressive than those of cells from passage 13 (one-tail paired *t*-test; *P* = .007,   *n* = 10).

In three of the cell preparations tested, the immunosuppressive activity was unchanged over the passages tested (single factor ANOVA; *P* = .064,   *n* = 6) (Figure 5(b)) while in two preparations the activity exhibited a continued decrease over time (single factor ANOVA; *P* = .017,   *n* = 4) (Figure 5(c)).

### 3.6. Effect of FGF-2 Supplementation on the Immunosuppressive Potential of hMSCs

**Figure 6:**
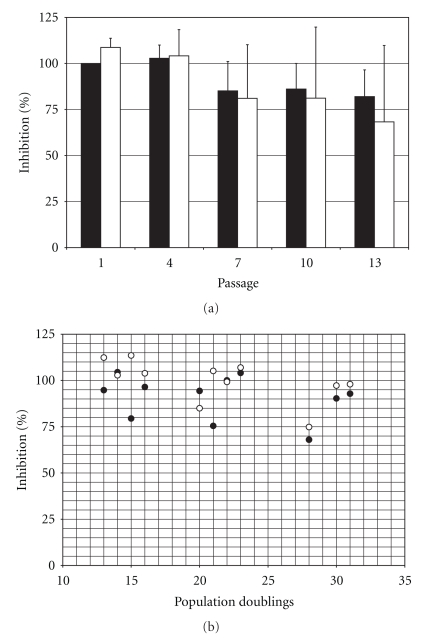
Effect of culture conditions on the immunosuppressive activity of hMSCs. Immunosuppressive activity of medium conditioned by IL-1*β*-stimulated hMSCs expanded in control or FGF-supplemented medium. Values are normalized to the inhibitory activity of first passage hMSCs expanded in control conditions. (a) Immunosuppressive activity (mean ± standard deviation; *n* = 5) of medium conditioned by IL-1*β*-stimulated hMSCs from different passages expanded in control (black bars) or FGF-supplemented (white bars) medium. (b) Immunosuppressive activity of medium conditioned by IL-1*β*-stimulated hMSCs expanded in control (black circles) or FGF-supplemented (white circles) medium. The data are presented as pairs of preparation- and passage-matched hMSCs subsets. Note that for 8 of the 11 data pairs (73%) the conditioned medium from FGF-treated hMSCs is more immunosuppressive than that of their control counterparts. Overall, the differences in immunosuppressive activity between conditioned media from control and FGF-treated cells are significant (one-tail paired *t*-test; *P* = .002).

No significant differences were observed between the immunosuppressive activity of cell preparation- and passage-matched hMSCs grown under control conditions or supplemented with FGF-2 (one-tail paired *t*-test;  *P* = .285; *n* = 25) (Figure 6(a)). However, the comparison of passage-matched groups may not be biologically relevant. As indicated above, the number of population doublings that FGF-treated cells have undergone at any given passage is higher than those of their control counterparts. When the comparison between the two different culture conditions was performed matching the subpopulations within each cell preparation by the number of population doublings rather than by the number of passages, cells expanded in FGF-supplemented medium exhibited higher immunosuppressive activity than their control counterparts (one-tail paired *t*-test; *P* = .002; *n* = 11) (Figure 6(b)).

## 4. Discussion

Due to their immunosuppressive properties, MSCs are being tested clinically to treat GVHD and other autoimmune disorders [18, 46–49]. However, because of the low frequency of MSCs in the bone marrow [50], the clinical application of hMSCs to treat GVHD requires extensive *ex vivo* expansion to achieve the cell doses currently utilized for patient treatment. For example, for an 80-kg patient, one single hMSC injection at the lowest dose (10^6^ cells per kg of body weight) being tested in clinical trials would necessitate 8 × 10^7^ cells. In this dataset, that level of expansion would have been reached by the end of the fourth passage under control conditions or by the end of the first passage under FGF-supplemented conditions. If multiple injections and/or higher doses were required, the cell number needed for the entire course of treatment could be as high as 4 × 10^9^ cells. In the current experiments, this higher level of expansion would require 7 or 8 passages in control and 4 passages in FGF-supplemented conditions.

The data presented here confirm the value of expansion in FGF-supplemented medium [31, 51–53]. Specifically, compared to expansion in control conditions, FGF-2 supplementation would result, on average, in 3,500-fold increase in the number of hMSCs obtained by passage 7, 24,000-fold increase in hMSCs by passage 10, and 240,000-fold increase in hMSCs by passage 13. These differences can be critical in the clinical application of these cells, as FGF supplementation can expedite production of cells resulting in more rapid preclinical testing, characterization, and availability of clinical-grade hMSCs, thereby significantly impacting the clinical application of MSC therapies.

It is well known that hMSC preparations have significant proliferative potential [19] but their extensive expansion results in loss of differentiation potential, onset of senescence [21], and/or apoptosis [54]. Although reports in the literature provide ample support for the immunosuppressive activity of hMSCs [10, 13, 14, 17, 55–57], the fate of this activity as the cell preparations are expanded has not been studied in depth. To date, only one report indicates that the immunosuppressive activity of hMSCs does not appear to decrease through 6 or 7 passages, as measured *in vitro* [58].

Our studies included the standard expansion conditions as initially described by Haynesworth and colleagues in 1992 [59] and supplementation with FGF-2 which has been shown to have beneficial effects on the proliferative and differentiation potentials of MSCs [27–29, 31, 32, 60]. We expanded the cell preparations for 13 passages, a level of expansion at which most hMSC preparations have reached senescence [19]. In this expansion, control MSCs reached 26.3 ± 4.7 population doublings by passage 13, while FGF-treated cells reached 44.2 ± 3.9. Of note, FGF-treated hMSCs reached 28.0 ± 2.6 population doublings (the maximum expansion obtained with control cells) around passage 6 in approximately 5 weeks compared to the 9 weeks required to achieve that level of expansion in control conditions, a difference that might be crucial for the timely administration of the cellular product.

FGF-treated hMSCs exhibited immunosuppressive activity comparable to that of passage-matched controls and that of population doubling-matched controls. Thus, while FGF supplementation does not elicit the significant improvements that have been reported for the chondrogenic potential of hMSCs [31], maintaining the immunosuppressive activity is critical for the application to disease conditions associated with aberrant T-cell alloreactivity. 

On a the technical aspect and relative to assay optimization, our data corroborate previous reports indicating that, at least in part, the immunosuppressive activity of hMSCs is mediated through soluble factors [13, 55, 61–63] and that hMSCs likely need to be stimulated by their microenvironment to exert this activity [14, 63, 64]. We have shown that medium conditioned by MSCs activated with PBMCs is immunosuppressive. This observation allows the use of conditioned medium to characterize the activity of the hMSCs. To this end, conditioned media from different preparations and passages can be prepared and stored and then tested simultaneously against the same effector cells, allowing better comparison of the results.

Additionally, the immunosuppressive activity of conditioned media from hMSC activated with PBMCs or IL-1*β* was comparable, but media generated with IL-1*β* had higher activity and, importantly, less variability in immunosuppressive activity, supporting the use of IL-1*β* instead of PBMCs for activation of the hMSCs and simplifying the preparation of the conditioned media in order to standardize stimulation using a recombinant cytokine rather than uncharacterized cell preparations.

## 5. Conclusions

In summary, compared to control conditions, hMSC expansion in FGF-supplemented medium would potentially benefit the intended recipients of these cellular products by enabling the acquisition of equivalent numbers of cells in a significantly reduced timeframe or more cells in the same period of time without loss of their immunosuppressive activity.

However, the immunosuppressive activity as measured *in vitro* using IFN*γ* ELISpot assays was not universally preserved in all the cell preparations from individual donors. Therefore, caution must be exercised when significant expansion of the MSCs is desired or required. In these cases, the activity of the final cell population should be verified, and perhaps activity or potency minimums should be established as part of the release criteria for therapeutic application. In addition, these initial *in vitro *observations must be validated using *in vivo* models because the microenvironment could certainly affect the activity or performance of these cells [65].
